# Editorial: Surgical skills and continuing medical education in neurosurgery: past, present and future

**DOI:** 10.3389/fsurg.2025.1671142

**Published:** 2025-08-12

**Authors:** Sami Ridwan, Michael Schwake, Mario Ganau

**Affiliations:** ^1^ Department of Neurosurgery, Klinikum Ibbenbueren, Ibbenbueren, Germany; ^2^ Department of Neurosurgery, University Hospital Münster, Münster, Germany; ^3^ Department of Neurosurgery, Oxford University Hospitals NHS FT, Oxford, United Kingdom; ^4^ Nuffield Department of Clinical Neurosciences, University of Oxford, Oxford, United Kingdom

**Keywords:** simulation, surgery, operating theatre, surgical innovation, surgical simulation models, surgical laboratory, microsurgical laboratory, virtual reality

**Editorial on the Research Topic**
Surgical skills and continuing medical education in neurosurgery: past, present and future

Surgical skill training as well as continuing medical education evolved in an unprecedented pace over the past few years (1). Trends similar to those seen in neurosurgery have been pinpointed also in other surgical specialty, partly due to the tremendous technical advances in ubiquitous access to artificial intelligence, machine learning and virtual reality (2–4), but also due to the impact of black swan events like the COVID-19 pandemic. In fact, modern visualization technologies and alternative means of communication were already available at the end of the last decade, nonetheless they found their way into daily practice much faster from 2020 onwards due to the accelerated adoption of digital technologies in education and clinical practice (5–8). The aim of this Research Topic was to highlight the changes and improvements brought up in the neurosurgery arena by new technologies and techniques utilized for surgical skill training and continuing medical education. This editorial will summarize the findings of the articles included in this collection.

Gatam et al. reported on the feasibility of robotic navigation in single position oblique lateral spine (OLIF) surgery. In their retrospective review of 25 consecutive patients who underwent robotic-navigated insertion of intersomatic cage through the oblique lateral corridor, the authors found that robotic guidance offers substantial benefits, including reduced bleeding, fewer surgical complications, and shorter operative times. Similar technical advantages were identified in the systematic review conducted by Kerimbayev et al. on the literature regarding minimally invasive insertion of transforaminal lumbar interbody fusion (MIS-TLIF) in the elderly. In fact, the authors provided compelling evidence supporting the long-term efficacy of such surgical strategy, suggesting that embedding technological advancements in our surgical practice allows for an extension of the current indications to the most challenging scenarios, something that has also been proved in other spinal segments and for various pathological conditions (9–11).

Spending time in an anatomy laboratory is known to be the best way to prepare for those complex surgical procedures and this is the case in cranial, spinal and peripheral nerve surgery. However the cost of setting up a wet laboratory and the availability of fresh cadavers represents entry barriers to providing ubiquitous standards for anatomical teaching, to the point that among recent breakthrough in the acquisition and retention of surgical skills we should mention the use of virtual reality, 3D printed models and digital twins (12–14). A relevant article for all medical students, trainees and junior doctors interested in anatomical learning is the one submitted by Tianjun et al. who offered an interesting perspective on mnemonic techniques applicable to brachial, lumbar and sacral plexuses. To this regard, the work from Shi et al. is also interesting: the authors described the educational impact of 3D models integrating micro-vessels, skull, and brain tissue as teaching aid for microvascular anastomosis simulation training, and concluded that such approach to neurosurgical training yields significant advantages, such as enhanced learning effectiveness, high-fidelity simulation, cost efficiency, and customization capabilities.

As we have seen, this Research Topic demonstrated how technological aids could facilitate not only the execution but also the planning of neurosurgical cases, with the aim of reducing our complication rates. This was precisely the rationale why the case report written by Karamani et al. found space in the list of accepted articles. The discussion from that article provides food for thought which is particularly useful to highlight how important is to leveraging on the high-tech revolution of the past 10 years to prevent fatal complications. In fact, although rare complications are not negligible even in the most common neurosurgical procedures (such as burr holes drainage of chronic subdural hematomas) hence the suggestion to embed them in our standard operating procedures for neurotrauma centers (15, 16).

In summary, this Research Topic has garnered significant interest as shown by the enthusiastic impact achieved to date: 5,518 interactions, consisting of 3.354 article views, 826 downloads and 1,338 topic views, respectively. Such metrics indicate that this niche of translational research applied to surgical training is attracting the attention of our wide and multidisciplinary readership, thus we are hopeful that this Research Topic will become a valuable reference among the related body of literature.
